# CONVERSATIONS ON THE COSTS OF CAREGIVING: COMPARING CAREGIVERS’ AND FINANCIAL ADVISORS’ PERCEPTIONS

**DOI:** 10.1093/geroni/igac059.2109

**Published:** 2022-12-20

**Authors:** Alexa Balmuth, Julie Miller, Lauren Cerino, Adam Felts, Lisa D'Ambrosio, Joseph Coughlin

**Affiliations:** Massachusetts Institute of Technology, Cambridge, Massachusetts, United States; Massachusetts Institute of Technology, Cambridge, Massachusetts, United States; Massachusetts Institute of Technology, Cambridge, Massachusetts, United States; Massachusetts Institute of Technology, Cambridge, Massachusetts, United States; Massachusetts Institute of Technology, Cambridge, Massachusetts, United States; Massachusetts Institute of Technology, Cambridge, Massachusetts, United States

## Abstract

Costs of caregiving go beyond physical, mental, and emotional; the financial costs are astronomical. On average, family caregivers spend 26% of their income on caregiving (AARP 2021). As the need for family caregivers grows due to longer lifespans (AARP 2020), incorporating the topic of caregiving into financial planning for longevity conversations becomes increasingly necessary. Drawing on data from the MIT AgeLab Caregiver Panel, a research panel of over 1400 family caregivers, and from the MIT AgeLab Preparing for Longevity Advisory Network, a research panel of over 900 financial professionals, this presentation will describe mixed methods research findings that highlight caregivers’ and financial professionals’ attitudes and perceptions toward caregiving as a topic within client-advisor conversations. Results demonstrate that many caregivers wish they had financially planned for the costs of caregiving more than they did. However, caregivers rarely turn to their financial advisors for support; many perceive their value to be strictly financial and are unaware of their advisors’ ability to support them. Despite caregivers’ doubts, most financial professionals feel equipped and willing to have caregiving-related conversations with clients. Additionally, while over three-quarters of financial professionals reported making referrals to outside resources for caregiving-related support, some critical avenues – including social workers and therapists, support groups, and respite care providers – were underutilized. Implications of these findings for caregivers and professionals of various industries will also be discussed.

